# Overuse of Chemical Restraints for the Treatment of Aggressive Psychiatric Inpatients : A Clinical Report

**DOI:** 10.1192/bjo.2026.11583

**Published:** 2026-06-30

**Authors:** Hajra Tariq, Mahnoor Irshad, Fahaddis Ahmed Rana, Sumira Alam, Nasar Sayeed Khan

**Affiliations:** 1 Services institute of medical sciences, services hospital, Lahore, Pakistan; 2 Queen's university, ontario, Canada

## Abstract

**Aims:**

Aggression in psychiatric inpatients is a frequent challenge that threatens staff safety and patient well-being. Although guidelines recommend verbal de-escalation as the first-line approach, restrictive practices such as chemical and physical restraints are often overused.

**Methods:**

A retrospective review of patient records was conducted at the Services Hospital Lahore psychiatry ward (January–June 2022). Data were compared against NICE NG10 and Maudsley prescribing guidelines.

**Results:**

A total of 30 inpatients were included (66.7% male, mean age 35.4 ± 11.6 years). Diagnoses included substance-related disorders (50%), schizophrenia/psychosis (40%), mood disorders (10%), and dissociative disorders (10%). All patients (100%) were managed with chemical restraints as first-line intervention; 20% also required physical restraints. None received verbal de-escalation first. Reasons included fear of patient violence (40%), rapid availability of medication (40%), and lack of staff training (20%).

**Conclusion:**

There is systemic over-reliance on chemical restraints in this setting, with minimal use of evidence-based de-escalation. Staff training and guideline adherence are urgently required.

